# The human bitter taste receptor T2R38 is broadly tuned for bacterial compounds

**DOI:** 10.1371/journal.pone.0181302

**Published:** 2017-09-13

**Authors:** Christophe Verbeurgt, Alex Veithen, Sébastien Carlot, Maxime Tarabichi, Jacques E. Dumont, Sergio Hassid, Pierre Chatelain

**Affiliations:** 1 Department of Otorhinolaryngology, Erasme University Hospital, Free University of Brussels, Brussels, Belgium; 2 ChemCom S.A., Brussels, Belgium; 3 Institute of Interdisciplinary Research in human and molecular Biology, Free University of Brussels, Brussels, Belgium; Scripps Research Institute, UNITED STATES

## Abstract

T2R38 has been shown to be a specific bacterial detector implicated in innate immune defense mechanism of human upper airway. Several clinical studies have demonstrated that this receptor is associated with the development of chronic rhinosinusitis (CRS). T2R38 was previously reported to bind to homoserine lactones (HSL), quorum sensing molecules specific of *Pseudomonas Aeruginosa* and other gram negative species. Nevertheless, these bacteria are not the major pathogens found in CRS. Here we report on the identification of bacterial metabolites acting as new agonists of T2R38 based on a single cell calcium imaging study. Two quorum sensing molecules (Agr D1 thiolactone from *Staphylococcus Aureus* and CSP-1 from *Streptococcus Pneumoniae*) and a list of 32 bacterial metabolites from pathogens frequently implicated in CRS were tested. First, we observed that HSL failed to activate T2R38 in our experimental system, but that the dimethylsulfoxide (DMSO), used as a solvent for these lactones may, by itself, account for the agonistic effect previously described. Secondly, we showed that both Agr D1 thiolactone and CSP-1 are inactive but that at least 7 bacterial metabolites (acetone, 2-butanone, 2-pentanone, 2-methylpropanal, dimethyl disulfide, methylmercaptan, γ-butyrolactone) are able to specifically trigger this receptor. T2R38 is thus much more broadly tuned for bacterial compounds than previously thought.

## Introduction

The taste type 2 receptors (T2Rs or Tas2Rs) receptors correspond to a subfamily of GPCRs initially found to be expressed in vertebrate’s tongue and to be dedicated to the perception of bitterness in food [1–3]. Recently, the expression of T2Rs has also been reported in different cell types and in different parts of the body such as airways, gastrointestinal tract, pancreas, white blood cells, heart, breast, thyroid, skin, testes [4]. The physiological relevance of these extra-oral expressions of T2Rs remains largely uninvestigated. However, recent studies provided evidences for the role of some of these receptors in the detection of bacterial products, more particularly in the upper airways. T2Rs were first detected in solitary chemosensory cells in the mouse nasal mucosa and the application of bitter compounds or bacterial quorum-sensing molecules on the nasal mucosa activate protective reflexes through stimulation of the trigeminal nerve while decreasing the respiratory rate [5,6]. Bacterial quorum-sensing molecules can also modify innate airway defense mechanisms in rat [7]. In human lower airways, T2Rs are located on motile cilia of epithelial cells and their stimulation increases ciliary beat frequency [8]. Therefore, this class of receptor constitute a potential target for the management of airway diseases.

Chronic rhinosinusitis (CRS) is an important public health issue with significant repercussions on patient’s quality of life and a substantial socioeconomic impact [9]. The pathophysiology of CRS remains unknown, but host-microbial interactions seem to play a leading role [10]. Several data demonstrate the implication of some T2Rs in CRS. In humans, a bitter taste receptor (T2R38) present in the sinus epithelium was reported to detect acyl homoserine lactone [11], a specific metabolic product of *Pseudomonas Aeruginosa* and other gram negative species that plays a role in the bacterial quorum sensing [12,13]. The activation of T2R38 drives NO production, leading to an increased mucociliary clearance and to direct antibacterial effects [11]. T2R38 have two major haplotypes, a functional (*PAV*) and a non functional (*AVI*), underlying taste sensitivity to phenylthiocarbamide [14,15]. CRS-affected patients bearing the recessive homozygous genotype have more chance to suffer sinonasal gram-negative bacterial infection [11] and require sinus surgery more frequently [16]. On another hand, patients affected by polyp free CRS who share the dominant homozygote status (*PAV*/*PAV*) for T2R38 have better surgical outcome after sinus surgery than the other genotypes [17]. Moreover, a genomic study involving several hundreds of patient has identified a significant association between a T2R38 coding SNP (I296V) and CRS. T2R38 functionality was further associated with sinonasal symptoms in healthy adults [18] and in cystic fibrosis patients [19]. All these data support the implication of T2R38 in CRS.

Since the causal agents of chronic rhinosinusitis are not limited to *Pseudomonas Aeruginosa* or even to gram negative bacteria, it is of interest to determine whether T2R38 can play the role of a broader detector of bacterial compounds. In this study, two quorum sensing molecules, from *Staphylococcus Aureus* and *Steptococcus Pneumoniae*, and a list of 32 bacterial compounds from pathogens implicated in rhinosinusitis were tested as potential agonists of T2R38, using an in vitro calcium-based functional assay.

## Material and methods

### Plasmid construction and cell line generation

The coding sequences of either the functional (*PAV*, GenBank accession number AY258597.1) and non-functional (*AVI*, GenBank accession number AF494231.1) were amplified from human genomic DNA by polymerase chain reaction.

These coding sequences were cloned in a pEFIB backbone vector in translational fusion with the coding sequence of a leader sequence corresponding to the 45 first amino acids of the rat somatostatin receptor subtype 3 [20]. The constructs were verified by sequencing. The coding sequence of RTP4 (Genbank accession number AY562238.1) was amplified from human spleen cDNA and cloned a pCI backbone vector.

The G_α16gustducin44_ sequence was generated by swapping the last 132 nucleotides of the G _α16_ with the corresponding terminal sequence of the G_α-gustducin_ [21] and was cloned in a pIRES-Puro plasmid. This vector was transfected in the Peakrapid cell line (ATCC reference: CRL-2828™; purchased from LGC Standards SARL, Molsheim, FRANCE), HEK293 cell line derivative, cultured in EMEM culture medium (reference BE12-136F; Lonza, Vervier, BELGIUM) supplemented with 10% of foetal bovine serum (Sigma-Aldrich, Bornem, Belgium) and 1 μg/ml of Puromycin to allow the selection of recombinant cell populations. Cell clones expressing the G_α16gustducin44_ were selected and the clone presenting the best functional response of T2R38 to phenylthicarbamide was used for the study (see below).

### Single cell calcium imaging

The Peakrapid cell line expressing stably a chimeric G_α16gustducin44_ G protein [21] was used. 40 000 cells were seeded in each well of a black/clear poly-D-lysine cellware pre-coated 96-well plate (Biocoat^®^, Becton-Dickinson, Erembodegem, Belgium) and transiently transfected with 50 ng of plasmid DNA containing the receptor sequence or not (empty vector) and with 50 ng of chaperone protein RTP4 per well. Forty hours after transfection, cells were incubated with the calcium-sensitive dye Fluo4 (Life Technologies, Oregon, USA) solubilized at 10 μg/ml in a buffer containing EMEM (150 mM NaCl, 5 mM KCl, 2 mM CaCl2, 1.2 mM MgCl2, 10 mM glucose), 10 mM HEPES, 0.01% (v/v) Pluronic F127, 0.1% (w/v) bovine serum albumin. After 1 h incubation at 37°C, cells were rinsed twice with the same buffer without Fluo4 and left in 50 μl buffer (prior ligand injection). Measurement were performed on a platform based on a motorized Axiobserver Z1 fluorescence microscope piloted by a Axiovision software (Zeiss, Jena, Germany) as described previously [22]. For each selected well, images were taken 1 per second, during 30 seconds. 50 μl of the buffer containing the ligand twice concentrated was injected on the 2nd second of the time lapse record.

After time lapse record, the number of responding cells in each injected well was determined using ImageJ 1.34 (Rasband, W.S., ImageJ, National Institutes of Health, Bethesda, Maryland, USA, http://rsb.info.nih.gov/ij/1997-2004), and expressed as the percentage of the surface of a microscope field covered by activated cells with respect to the total surface of the field covered by confluent cells. Automated determination of the surface occupied by responding cells is easier to perform than a cell counting. The resulting percentage of positive surface provided a convenient estimation of the percentage of responding cells, as observed by comparing the percentage of positive surface and percentage of responsive cells determined by direct counting, in the same experiment. In agreement with the literature [23], the percentage of responding cells was proportional to the agonist concentration. During experiment in each plate, at least 2 wells were tested with phenylthiocarbamide (PTC) at 0.1 mM, which is one of the best characterized agonist of T2R38 in the literature. The activation of T2R38 by PTC allows normalization of the result of other tested molecules. The results were thus expressed as the percentage of PTC activation. In scaling calcium imaging assays using this latter receptor, concentration-responses curves were performed and data were fitted to a four parameter logistic equation: $y=\text{min}+\;\frac{\left(\text{max}-\;\text{min}\right)}{1+10{\;}^{(\text{log}EC50-x)nH}}$. Min and max are the minimum and the maximum values of the percentage of cell-activated surface respectively, *y* is the percentage of cell-activated surface at the *x* concentration and *nH* is the Hill coefficient. While setting up the assay, concentration-response curves with PTC were performed, yielding to an EC50 value of 9.7 ± 0.4 μM (mean ± SEM of n = 3 experiments) (S2 Fig), similar to Behrens et al [24].

The results were confirmed by three independent experiments.

### Bacterial compounds

Two quorum-sensing molecules respectively from *Staphylococcus Aureus* and *Steptococcus Pneumoniae* were used: Agr D1 thiolactone (YSTc(CDFIM)) from Eurogentec (Seraing, Belgium) and CSP-1 (competence stimulating peptide -1) (EMRLSKFFRDFILQRKK) from Cellmano Biotech Limited (Hefei, China).

To reproduce results from the literature [11], two others quorum-sensing molecules from gram negative bacteria, N-butyryl-L-homoserine lactone (C4HSL) and N-3-oxo-dodecanoyl-L-homoserine lactone (C12HSL), were purchased respectively from Cayman Chemichal (Tallinn, Estonia) and from Sigma—Aldrich (Schnelldorf, Germany).

32 volatile metabolites of pathogens (S1 Fig) were selected from the literature [25–28]. All compounds were dissolved in buffer solution, except C4HSL dissolved in buffer solution, DMSO or ethanol, C12HSL dissolved in DMSO or ethanol, and Agr D1 thiolactone dissolved in DMSO.

All compounds were tested first at a high concentration (100 mM). If the result was non specific, lower concentration (1:2 dilution) were tested until a specific or no calcium response was found.

### Statistical analysis

To compare the levels of activation of T2R38 experiments and the controls, Mann-Whitney U tests were computed in R v3.2.3. P-values were then adjusted to account for multiple testing, using the function p.adjust with method = "fdr" to compute the false discovery rates.

## Results

### DMSO is an agonist of T2R38 and questions the agonistic effect of C4HSL and C12HSL

**Fig 1:**
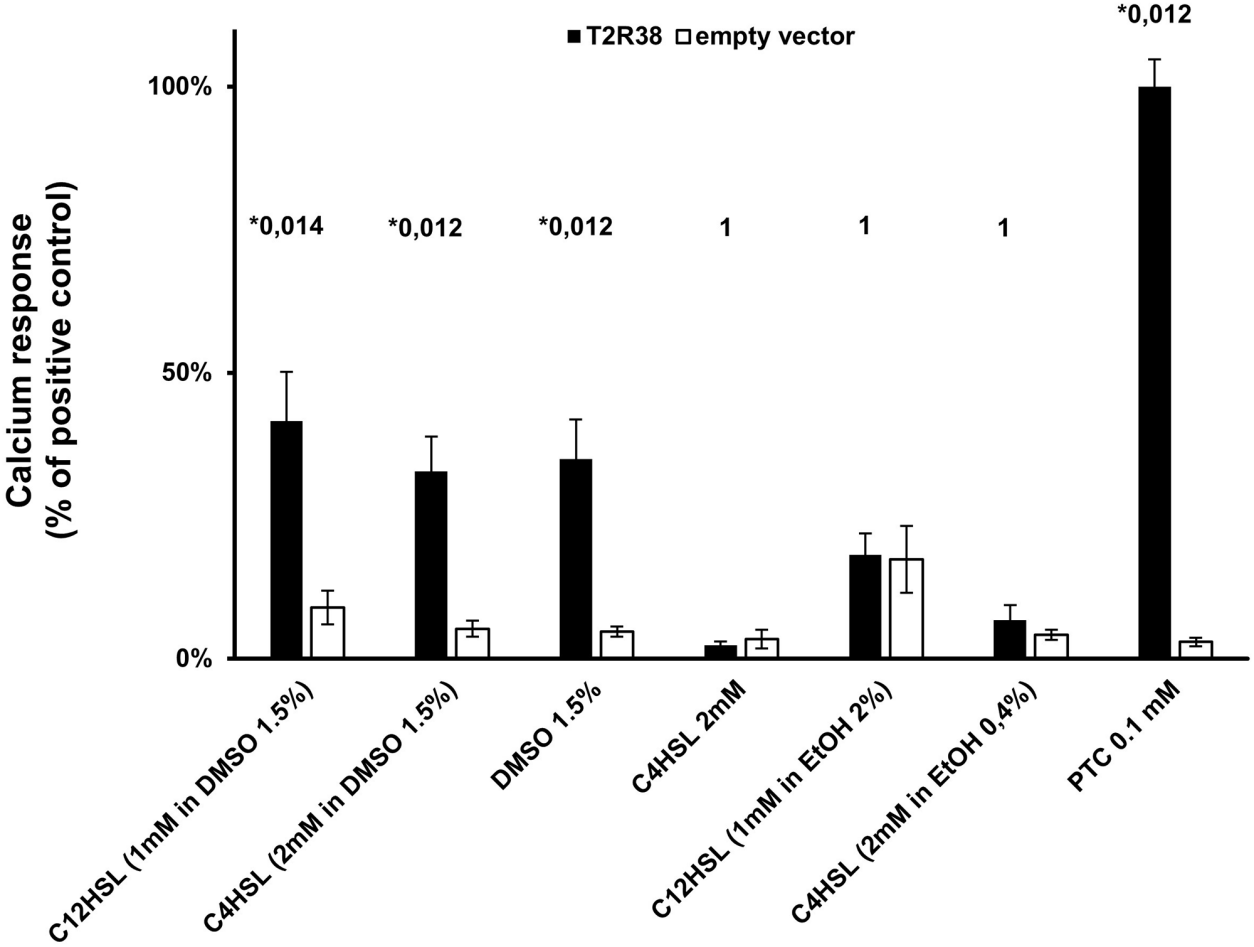
DMSO is an agonist of T2R38 which questions the agonistic effect of N-butyryl-L-homoserine lactone (C4HSL) and N-3-oxo-dodecanoyl-L-homoserine lactone (C12HSL). The response of T2R38 to a stimulation by quorum sensing lactone and to the solvent dimethylsulfoxide (DMSO) was monitored using a Ca^2+^-based functional assay. Results are presented as the percentage of the response elicited by 0.1 mM of phenylthiocarbamide (PTC) used as a reference agonist. Dimethylsulfoxide (DMSO) activated T2R38. Controls correspond to mock transfected cells. Results are presented as mean ± SEM of 3 independent experiments. The false discovery rates (fdr) are indicated above the columns, * indicate a significant result (fdr < 0.05).

**Fig 2:**
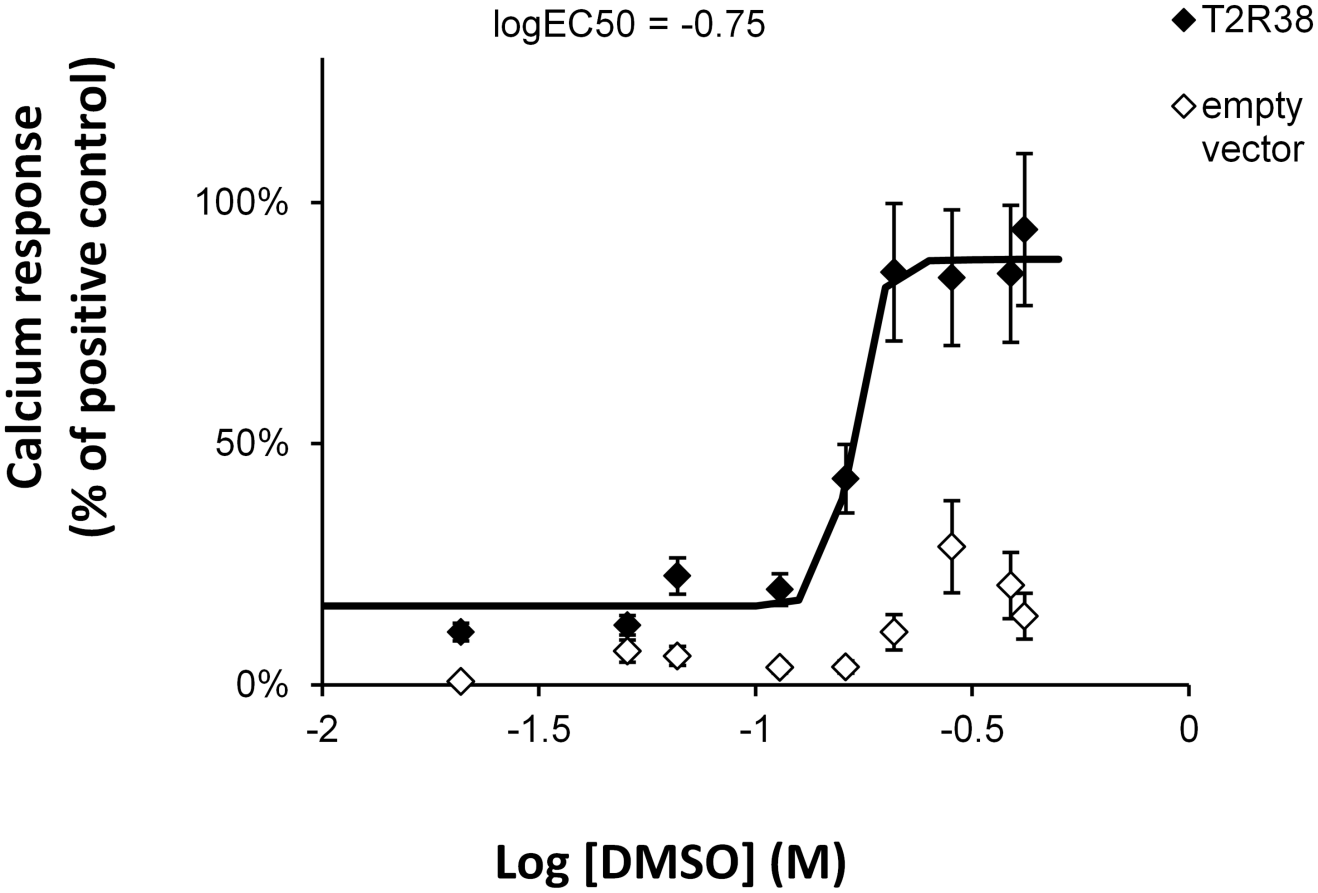
Concentration response curve of Peakrapid cells expressing T2R38 after stimulation with increasing DMSO (EC50 = 178 mM). Results are presented as the percentage of the response elicited by 0.1 mM of phenylthiocarbamide (PTC) used as a reference agonist. Controls correspond to mock transfected cells. Results are presented as mean ± SEM of 3 independent experiments.

With the intent of exploring the receptivity of T2R38 for bacterial products, we first tried to reproduce previous results showing that C4HSL and C12HSL activate specifically T2R38 [11]. Our experimental system relied on a transient expression of the receptor Peakrapid cells (a derivative of HEK293 cell line) that stably expressed the chimeric protein Gα16gus44. This model allows an efficient coupling of bitter taste receptors activation to an IP3-dependent increase of cytosolic calcium increase [21]. Regarding the poor water solubility of C4HSL and C12HSL, we used DMSO as a solvent as described by Lee and coworkers [11]. When dissolved in DMSO 1.5% (209 mM), C4HSL and C12HSL elicited an average calcium response of respectively 33% and 42% of the one induced by 0.1 mM of PTC used as a positive control (Fig 1). To verify the specificity of the response, the vehicle solution alone was also tested. Unexpectedly, DMSO 1.5% (209 mM) alone induced a T2R38-dependent calcium response of the same intensity. A lower dilutions of 0.1 mM of C12HSL with either 0.3% or 0.15% percent of DMSO in the incubation buffer, did not trigger any specific activation of the receptor (S3 Fig). This surprising observation seriously questioned of the ability of HSLs to trigger the receptor in our experimental system and clearly contrasted with the results of the inspiring study of Lee et al. [11]. Indeed, it showed a calcium response elicited by both C4HSL and C12HSL in primary culture of sinus epithelial cells from patients homozygote for the active T2R38 (PAV) allele but not from patients heterozygote or homozygote for the inactive (AVI) allele. The triggering of a T2R38-mediated calcium response upon stimulation with the HSLs was also observed in HEK293 cells expressing G16gus44, i.e. an experimental system equivalent to ours. Therefore, we tried alternative modes of solubilization of the lactones. C4HSL can be solubilized in the aqueous buffer, but did not elicit a response of T2R38 when assayed at 2 mM, i.e. the same final concentration than used when solubilized in DMSO. Likewise, ethanol-solubilized C4HSL and C12HSL did not trigger a specific activation of T2R38. These results indicate that C4HSL and C12HSL are not agonists of the receptor in our hands. Relying on the description of HSLs solubilization (stock solution at 1:1,000 in DMSO) in the publication of Lee et al [11], we calculated that the amount of DMSO introduced in experiments might reach up to 3% (422 mM) or more, i.e. a concentration that could fully account for the activation of T2R38. This first conclusion is further supported by the concentration-response analysis performed with DMSO on T2R38 (Fig 2). It demonstrated an EC_50_ value of 178 mM. The images of the calcium response are shown in the supplementary data (S4 Fig). The specific interaction of DMSO with T2R38 is also illustrated by the absence of effect of this molecule on the non-functional haplotype of T2R38 (*AVI*) (S5 Fig).

### Several bacterial metabolites activate T2R38

Published data suggest a role for T2R38 in chronic rhinosinusitis and bacterial detection [11]. In spite of our first results showing that C4HSL and C12HSL are not agonists for this receptor, we have not definitively dismissed this hypothesis and have considered other bacterial molecules as putative activators of the receptor.

First, two quorum-sensing molecules of bacterial species implicated in rhinosinusitis were tested on T2R38 (S6 Fig). Agr D1 thiolactone (from *Staphylococcus Aureus*) was not soluble in aqueous solution and therefore solubilized in 0.3% DMSO at a final concentration of 0.1 mM. This concentration did not show specific activation. CSP-1 (from *Streptococcus Pneumoniae*) was soluble at 0.5 mM in the buffer solution. The calcium response observed upon stimulation with concentrations between 0.2 to 0.5 mM was similar in both T2R38-expressing cells and in negative control, avoiding to conclude to a specific activation of the receptor.

**Fig 3:**
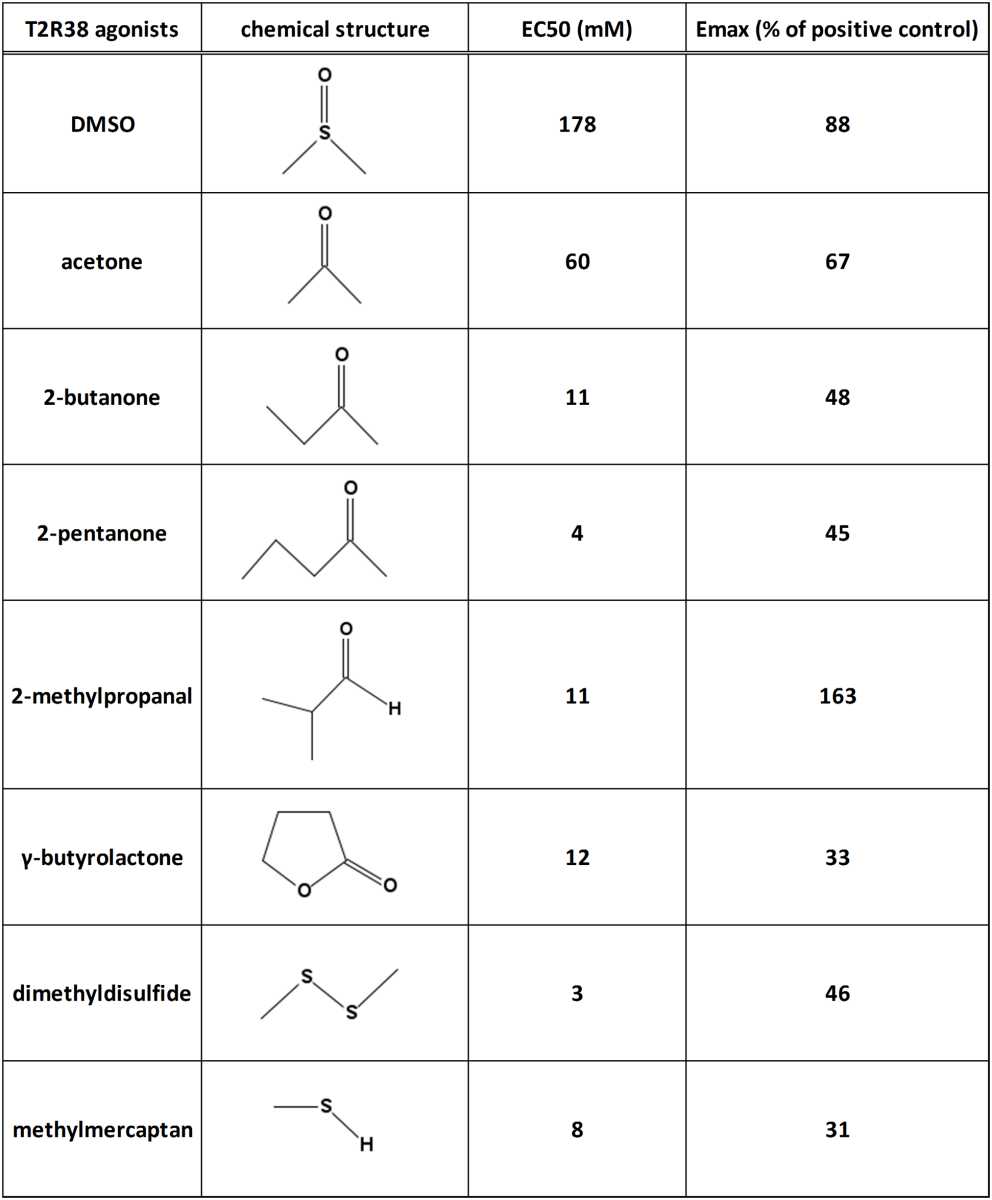
List of the 8 new T2R38 agonists. Emax is the percentage of the response elicited by 0.1 mM of phenylthiocarbamide (PTC) used as a reference agonist.

**Fig 4:**
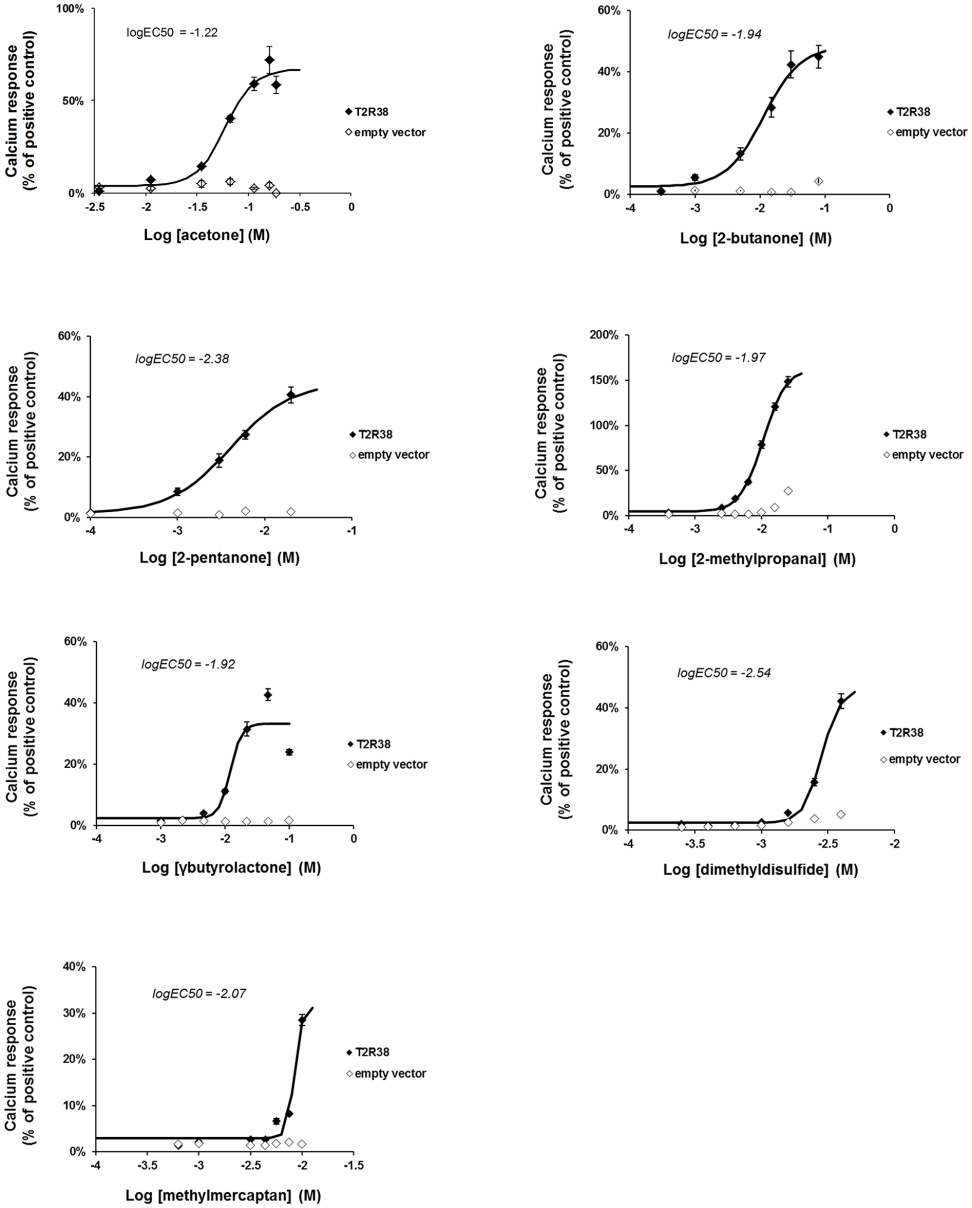
Activation of T2R38 by different bacterial metabolites. Results are presented as the percentage of the response elicited by 0.1 mM of phenylthiocarbamide (PTC) used as a reference agonist. Controls correspond to mock transfected cells. Results are presented as mean ± SEM of 3 independent experiments.

Secondly, it was of interest to determine whether common bacterial metabolites can activate T2R38. 32 volatile metabolites of pathogens (S1 Fig) were selected on the basis of the implication of the corresponding pathogen in rhinosinusitis, the level of abundance in bacterial medium [25–28] and the purchase availability. Over these 32 selected compounds, 7 produced an activation of T2R38 (Figs 3 and 4 and S7).

It is striking to note that none of the alcohols, carboxylic acids, esters or nitrogen bearing compounds are active. To date, 21 ligands have been reported for T2R38 [29,30]. They are characterized by a high degree of diversity of unrelated chemical structures and different potencies. The most potent agonists are active at low concentrations, below 100 μM whereas the less active need to be used at more than 1 mM to trigger the receptor [29], i.e. in the same range than the agonists identified in the present study. None of them are acids in line with our data. Known ligands of T2R38 have molecular weight and cLogP values ranging from 470 to 89 and +4.04 to -2.71 respectively. The compounds identified in our study have cLogP values from +1.39 to -1.28 within the range of the known ligands and molecular weights from 94 to 48 overlapping with the range of the known ligands and extending to the low molecular weight.

## Discussion

The application of bitter compounds on airways leads to various effects, such as bronchorelaxation or triggering of innate immune defenses. Recent studies have pinpointed the involvement of T2Rs in these mechanisms. More precisely, T2R38 was found to be expressed in sinonasal epithelial cells and to be activated by HSLC4 and HSLC12, two quorum sensing molecules secreted by *Pseudomonas Aeruginosa* and other gram negative bacteria. An increase of mucociliary clearance and NO production of the upper airway epithelium would result from the triggering of this receptor [11].

In the present study, we failed to reproduce the activation of T2R38 by HSLC4 and HSLC12 in a heterologous HEK293 cell-based expression system but we observed that the receptor responds to DMSO and to other bacterial metabolites than HSLC4 and HSLC12. These results are in apparent contradiction with the conclusions of the above mentioned study of Lee and coworkers [11]. A deeper analysis of the experimental conditions reveals that HSLC12 and HSLC4 used by Lee et al in single cell calcium imaging experiments were solubilized in DMSO.

According to the Method section of the corresponding publication, HSLC12 and HSLC4 were first prepared at 1:1,000 stock solutions in DMSO and used at 100 or 200 μM. In this condition, we calculated that the final concentrations of the solvent reached 3% (for HSLC12 at 100 μM) and 3.24% to 6.48% (for HSLC4 at 100 and 200 μM respectively). At these concentrations, DMSO alone could fully account for the observed activation of the receptor. Control experiments presented in the study of Lee et al [11], showed no effect of DMSO at concentrations up to 1%, in accordance with our own results. However, a possible activation at higher concentrations in DMSO was not provided. During the course of the reviewing of our manuscript, one of the co-author of Lee et al [11] stated that HSLC12 and HSLC4 where first solubilized at 100 mM before being diluted 1:1,000 (an additional information that cannot be read in the publication), making the concentration of DMSO at 0.1% i.e, below the activation threshold. Nevertheless, our observations that ethanol-solubilized HSLC4 and HSLC12 and water-solubilized HSLC4 remained without effect on the receptor are still in contradiction with an activation of T2R38 by HSLs. However, the results obtained with heterologous expression system we used does not rule out the possibility of an interaction of T2R38 with HSLs. Indeed, the main part of the work of Lee et al, was performed on primary culture of sinus epithelial cells. Peculiar phenomena of heterodimerization, coupling with alternative G protein or signaling cascade, as well as biased agonism could occurred in this cells that would be missed by our experimental system where a single receptor is expressed and the coupling to a chimeric G protein is forced. Additional indirect arguments in favor of an interaction T2R38 with HSLs are provided by the fact that culture supernatant of Pseudomonas aeruginosa culture increases NO secretion, ciliary beat frequency and bacteria killing by primary epithelial cells from individuals that expressed a functional T2R38 (PAV/PAV) but not from those expressing the non-functional allele (AVI/AVI) [11]. Interestingly, these effects are not observed with supernatant from a mutant strain, deficient for HSLs production. Other observations made in myeloid cells and neutrophils show that HSLC12 can bind T2R38 and this binding is inhibited by an antibody to T2R38. This binding is further illustrated by pull down assays using FITC-coupled or biotin-coupled HSLC12 [31, 32].

Although the heterologous expression system used here does not allow to study the whole range of T2R38 interactions, it remains a valuable tool for a pharmacological characterization of the receptor and enabled to discover the agonist status of DMSO. It is of particular importance since DMSO is a common solvent and its inappropriate use (i.e. high concentration) can drive to misleading interpretations while assessing potential agonist compounds on T2R38.

DMSO is known to have a bitter taste [33] but its potency as an agonist of T2R38 in the airway is particularly low as the active concentrations (EC50 = 178 mM) are far over those of bacterial products found in the airway. It is therefore unlikely that DMSO corresponds to a natural ligand that T2R38 would be assumed to detect in the sinus. Subsequently, we investigated the agonist potential of similar molecules. Replacing the sulfur atom by a carbon gives acetone that also activates T2R38 (EC50 = 89 mM). A further lengthening of the carbon chain results in 2-butanone and 2-pentanone, two additional stronger agonists of the receptor in our hands (EC50 = 11 mM and 4 mM, respectively). Interestingly, these two latter ketones are known bacterial metabolites produced by *Pseudomonas Aeruginosa* and other bacterial species [25].

The extension of our analysis to 32 additional volatiles metabolites of pathogens [25], selected on 1) the basis of the implication of the bacteria in rhinosinusitis, 2) the level of abundance in bacterial medium and 3) the purchase availability, led to the identification of 7 new agonists of T2R38. These molecules are not specific of a single bacterial species, but are produced by several pathogens including *Staphylococcus aureus*, *Pseudomonas aeruginosa*, *Streptococcus pneumoniae*, *Klebsiella pneumoniae*, *Escherichia coli* and *Enterococcus faecalis*, except maybe the 2-methylpropanal that is produced by *Staphylococcus aureus* but not by the other pathogens [25]. Based on the interaction of T2R38 with quorum sensing molecules of *Pseudomonas aeruginosa*, Lee and coworkers have proposed that this receptor plays a role in the triggering of innate immunity in sinonasal pathologies. Although the results presented here question the T2R38 agonist status of HSLC4 and HSLC12, our results are still supportive of this hypothesis. Since many clinical studies suggest an involvement of T2R38 in CRS [11,16,17,18,19], it was surprising that this receptor only detected product from bacteria less frequently implicated in this sinonasal pathology. Our results strongly suggest that T2R38 has a larger range of agonists from a bacterial origin and therefore, would be tuned to detect non-selectively a broader spectrum of bacteria, including *Staphylococcus aureus*, the most frequent pathogen in CRS [9].

T2R38 has been well characterized on a pharmacological point of view. At least 21 agonists have been identified so far [29,30] and are active in or below the millimolar range. The role of this receptor in the taste perception of several of these agonists has also been demonstrated by the correlation of a specific inability to taste the corresponding molecule with genetic alteration in the coding sequence of T2R38 [14,15]. It is well accepted that the main physiological role of bitterness perception consists in preventing the ingestion of toxics. It can therefore be conceived that T2Rs have been evolutionarily selected to detect those poisons at a low concentration.

The situation could be rather different when considering the role of a T2R in bacterial detection and triggering of innate immune defenses. This mechanism would serve in maintaining a stable microbiome on the sinonasal epithelium. Such system should be effective when the quorum of pathogenic bacteria reaches a critical threshold. The involved receptor would therefore be activated by bacterial metabolites at a concentration generated by a relatively dense population. Consistently, the active concentrations of the 7 bacterial metabolites reported here to agonize T2R38 are all over the millimolar range. So far, there is no report of accurate measurement of these metabolite concentrations in the sinusonasal mucus that could comfort our hypothesis. Nevertheless, in culture conditions, concentrations up to 10 mM have been reported. For example, 2-pentanone can be detected at concentrations exceeding 40 mM in *Aspergillus Niger* culture [34], 2-methylpropanal can reach 15 mM in *Cyanobacteria* culture [35] and methylmercaptan 100 mM in another bacterial culture [36]. Moreover, in an adherent film of maximal width of 100 μm on airway mucosa even small quantities could achieve such high concentration.

Our study presents several limitations. First, the results were obtained in a heterologous expression system using a single cell calcium imaging assay that may be far from physiological reality. It will be necessary to confirm the effect of the bacterial metabolites in more physiological conditions, like explants of sinonasal mucosa. Another limitation relies in the number of bacterial metabolites tested. We selected the molecules among a short list of volatile metabolites of pathogens. This is of course not comprehensive of all bacterial metabolites, and it would be worth extending this research to other released bacterial products. Likewise, we have limited our analysis to T2R38 because of the evidences provided in the literature of a role of this receptor in CRS pathogenesis. Other chemosensory receptors, bitter or olfactory, might also be involved since they are expressed in the airways [37–39]. Together, they could constitute an information network about bacterial populations.

## Supporting information

S1 Fig 32 volatile metabolites of pathogens tested. (TIF)

S2 Fig Concentration response curve with PTC. Controls correspond to mock transfected cells. Results are presented as mean ± SEM of 3 experiments (n = 6).
(TIF)

S3 Fig A lower dilutions of 0.1 mM of C12HSL with either 0.3% or 0.15% percent of DMSO in the incubation buffer, did not trigger any specific activation of T2R38. Results are presented as mean ± SD. The calcium response is relative to 100% T2R38 activation by 0.1 mM of PTC.
(TIF)

S4 Fig Pictures of calcium cell responses at 30 seconds after injection of DMSO. Cells were transfected with T2R38 plasmid (right column) and control experiments with an empty vector (left column).
(TIF)

S5 Fig DMSO 1.5% (209 mM) activates specifically T2R38. *PAV* is the functional allele of the receptor. Controls correspond to cells transfected with nonfunctional allele (*AVI*). Results are presented as mean ± SEM. The calcium response is relative to 100% T2R38 activation by 0.1 mM of PTC.
(TIF)

S6 Fig Response to CSP-1 (from *Streptococcus Pneumoniae*) was non specific. Agr D1 thiolactone (from *Staphylococcus Aureus*) did not activate T2R38. Results are presented as mean ± SD. Controls correspond to mock transfected cells.
(TIF)

S7 Fig Seven bacterial metabolites activate specifically T2R38. *PAV* is the functional allele of the receptor. Control is cell transfected with nonfunctional allele (*AVI*). Results are presented as mean ± SEM. The calcium response is relative to 100% T2R38 activation by 0.1mM of PTC. The false discovery rates (fdr) are indicated above the columns, * indicate a significant result (fdr < 0.05).
(TIF)

S1 Table Data reporting. (XLSX)
