# Supplementary figures and images for: The human bitter taste receptor T2R38 is broadly tuned for bacterial compounds
Source: PLoS One. 2017 Sep 13;12(9):e0181302. doi: 10.1371/journal.pone.0181302 (PMC5597121; doi:10.1371/journal.pone.0181302)

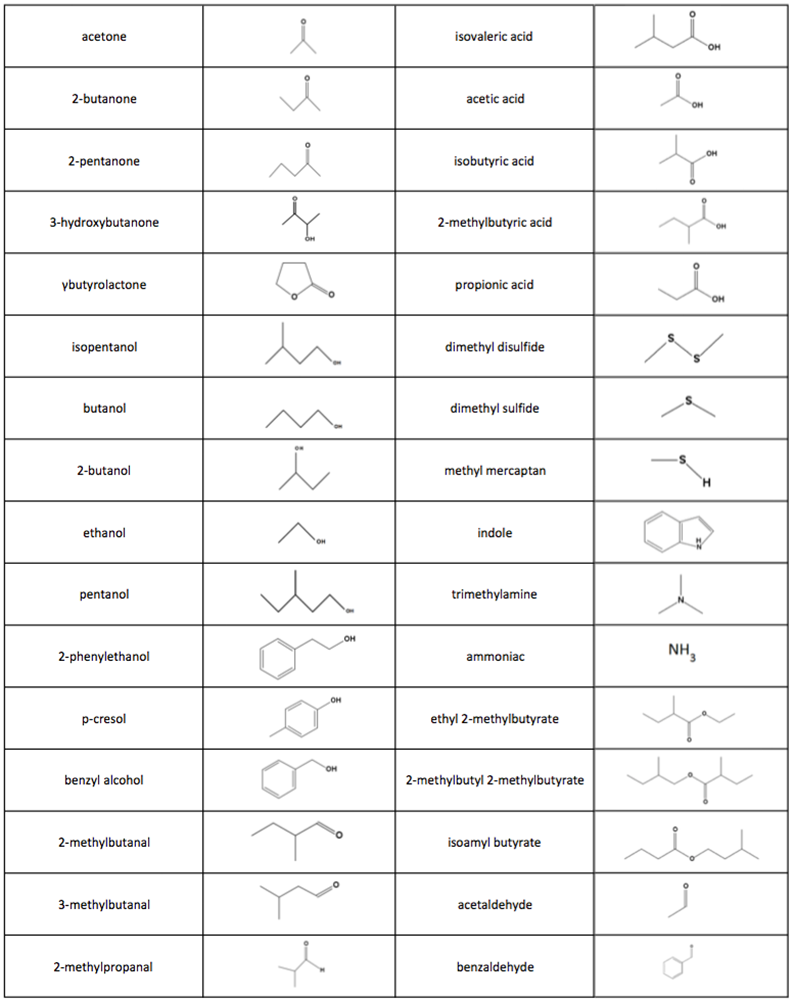

Supplement: S1 Fig — (TIF) [file pone.0181302.s001.tif]

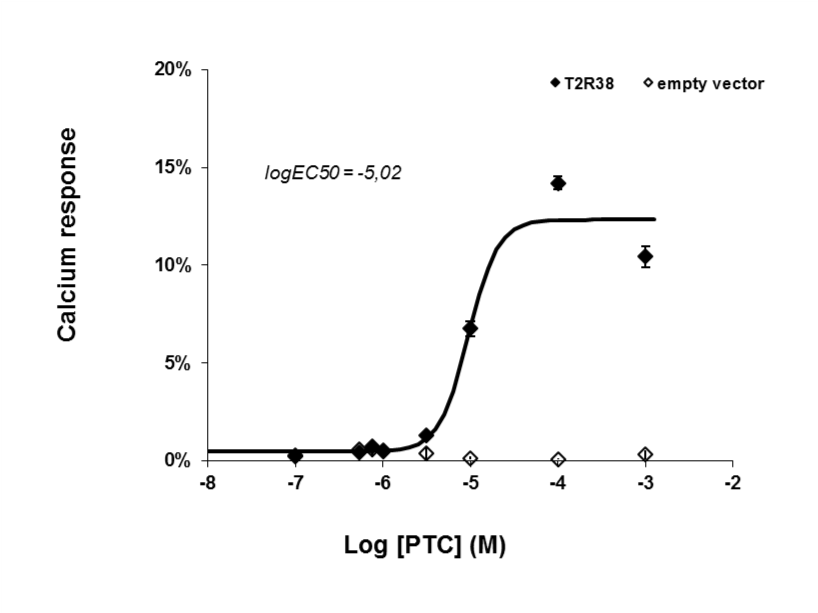

Supplement: S2 Fig — Controls correspond to mock transfected cells. Results are presented as mean ± SEM of 3 experiments (n = 6). (TIF) [file pone.0181302.s002.tif]

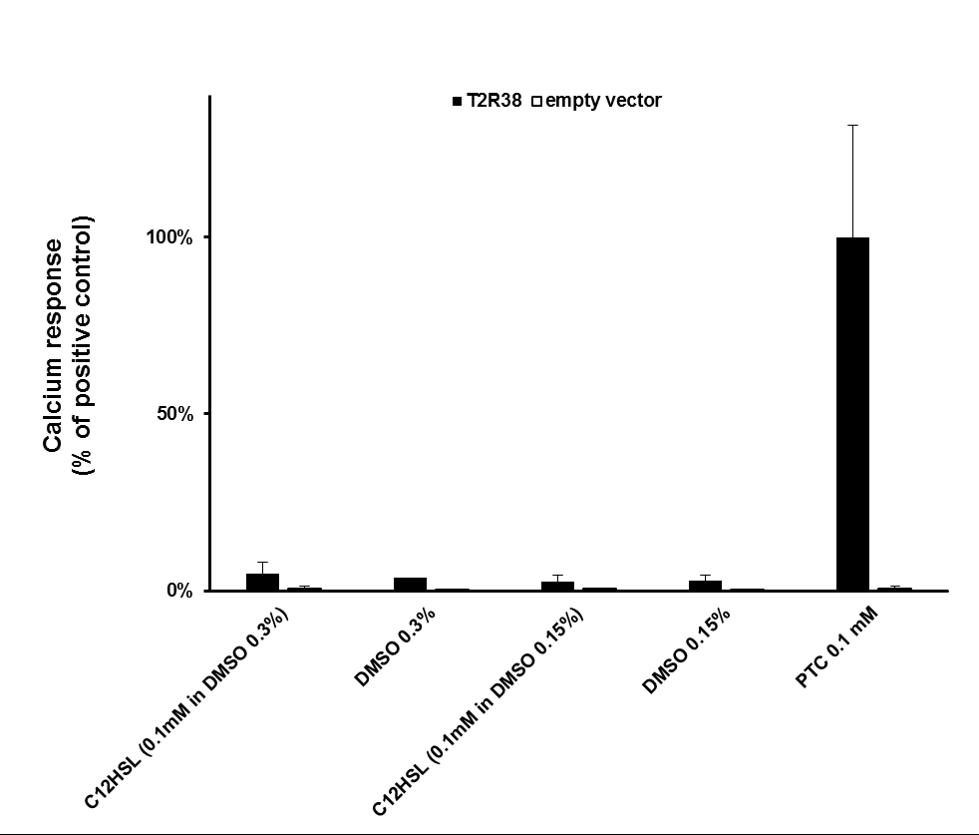

Supplement: S3 Fig — Results are presented as mean ± SD. The calcium response is relative to 100% T2R38 activation by 0.1 mM of PTC. (TIF) [file pone.0181302.s003.tif]

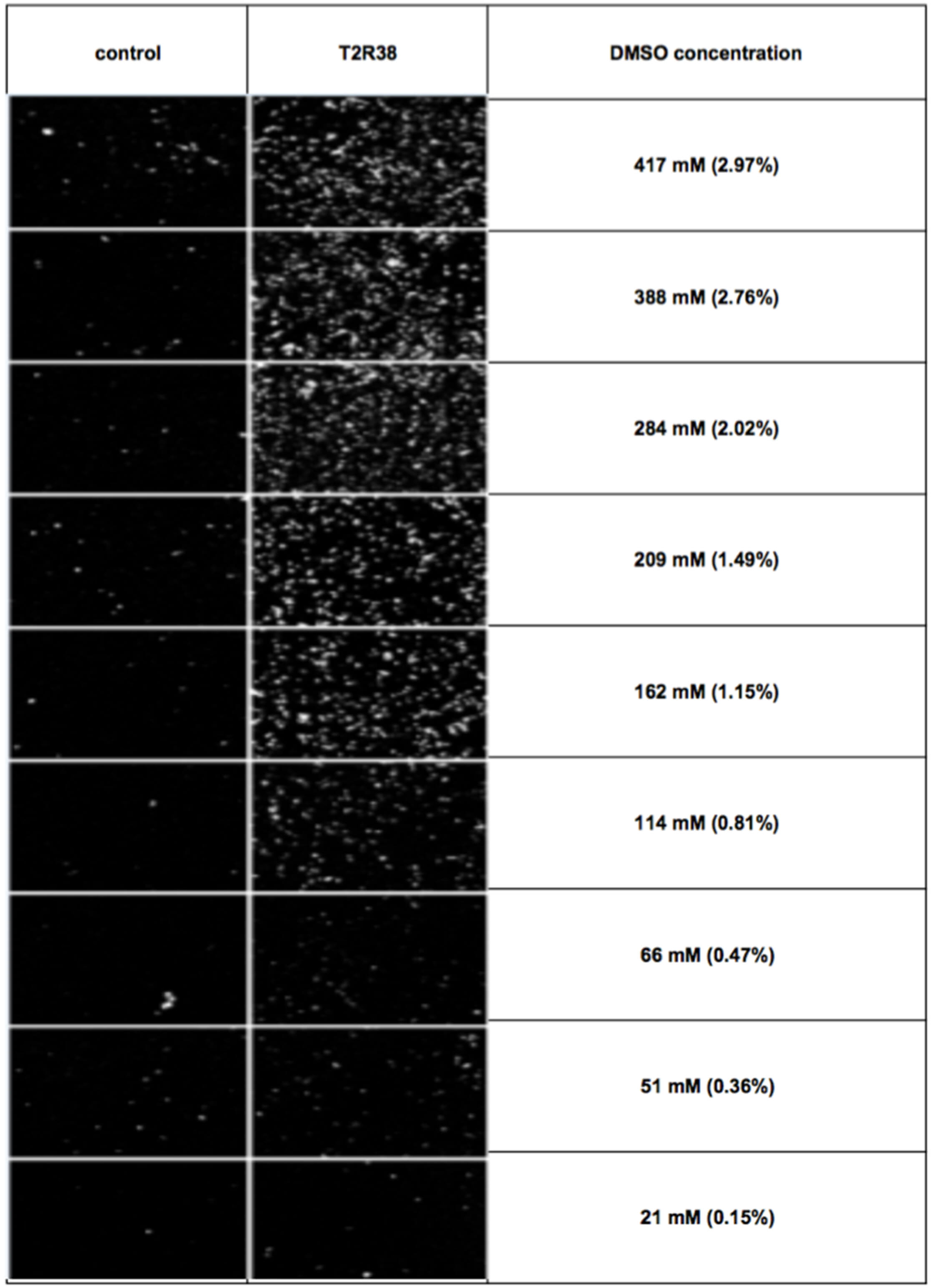

Supplement: S4 Fig — Cells were transfected with T2R38 plasmid (right column) and control experiments with an empty vector (left column). (TIF) [file pone.0181302.s004.tif]

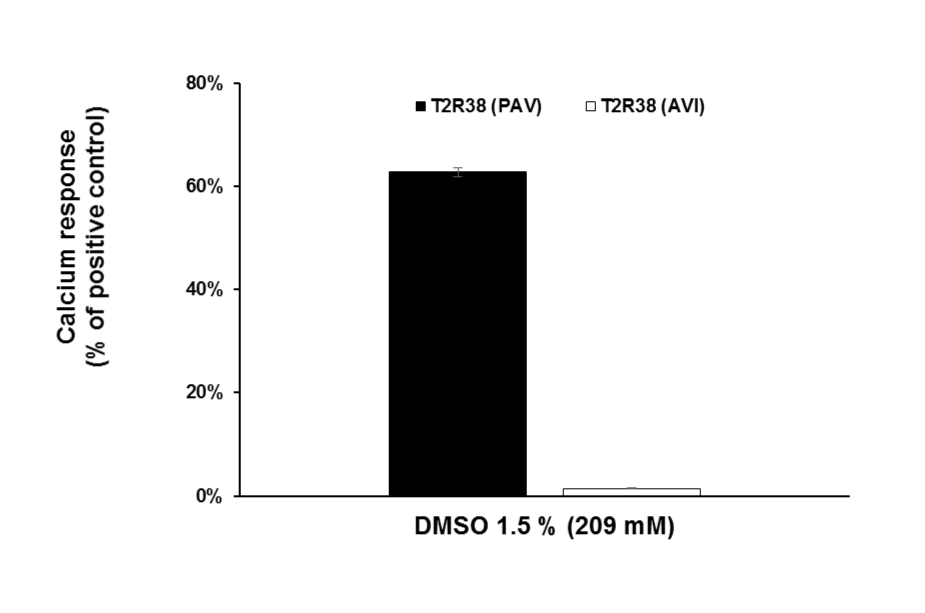

Supplement: S5 Fig — PAV is the functional allele of the receptor. Controls correspond to cells transfected with nonfunctional allele (AVI). Results are presented as mean ± SEM. The calcium response is relative to 100% T2R38 activation by 0.1 mM of PTC. (TIF) [file pone.0181302.s005.tif]

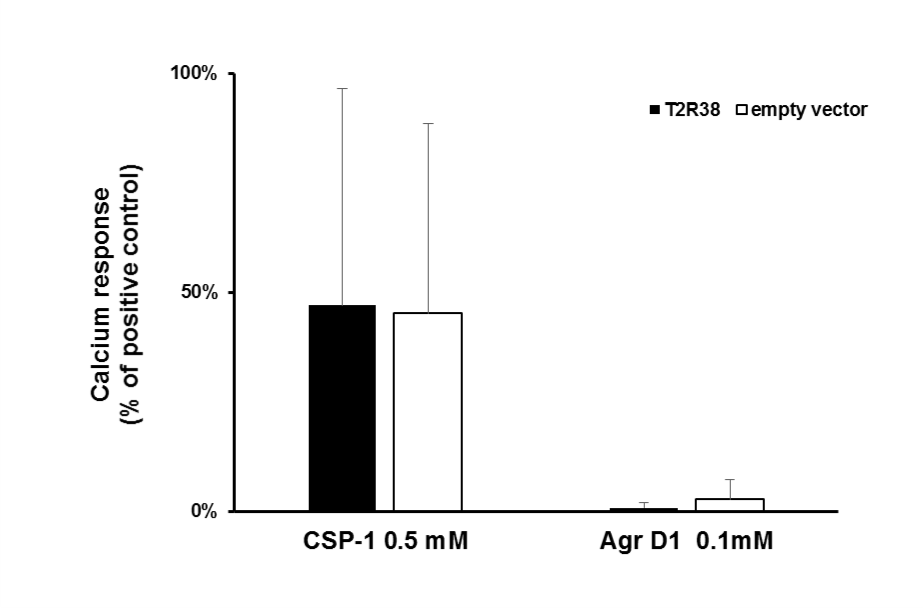

Supplement: S6 Fig — Results are presented as mean ± SD. Controls correspond to mock transfected cells. (TIF) [file pone.0181302.s006.tif]

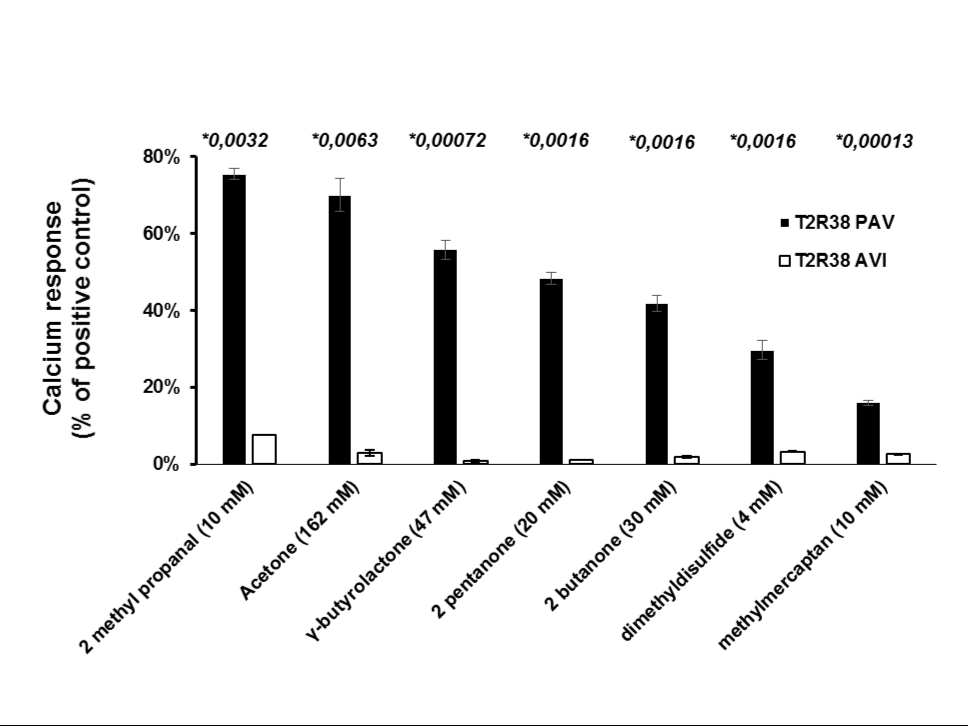

Supplement: S7 Fig — PAV is the functional allele of the receptor. Control is cell transfected with nonfunctional allele (AVI). Results are presented as mean ± SEM. The calcium response is relative to 100% T2R38 activation by 0.1mM of PTC. The false discovery rates (fdr) are indicated above the columns, * indicate a significant result (fdr < 0.05). (TIF) [file pone.0181302.s007.tif]
